# Visceral fat area is the measure of obesity best associated with mobility disability in community dwelling oldest-old Chinese adults

**DOI:** 10.1186/s12877-021-02226-6

**Published:** 2021-04-28

**Authors:** Kevin Yiqiang Chua, Xinyi Lin, Yeli Wang, Yap-Seng Chong, Wee-Shiong Lim, Woon-Puay Koh

**Affiliations:** 1 Integrative Sciences and Engineering Programme, NUS Graduate School, National University of Singapore, Singapore, Singapore; 2 Saw Swee Hock School of Public Health, National University of Singapore, Singapore, Singapore; 3 Centre for Quantitative Medicine, Duke-NUS Medical School, Singapore, Singapore; 4 Singapore Clinical Research Institute, Singapore, Singapore; 5 Health Services and Systems Research, Duke-NUS Medical School, Singapore, Singapore; 6 Department of Obstetrics & Gynaecology, Yong Loo Lin School of Medicine, National University of Singapore, National University Health System, Singapore, Singapore; 7 Singapore Institute for Clinical Sciences, Agency for Science Technology and Research (A*STAR), Singapore, Singapore; 8 Department of Geriatric Medicine, Institute of Geriatrics and Active Aging, Tan Tock Seng Hospital, Singapore, Singapore; 9 Healthy Longevity Translational Research Programme, Yong Loo Lin School of Medicine, National University of Singapore, Singapore, Singapore

**Keywords:** Body mass index (BMI), Waist circumference, Percent body fat, Visceral fat area, Bioelectrical impedance analysis (BIA), Locomotive syndrome, Mobility disability

## Abstract

**Background:**

Although obesity can be clinically defined by body mass index (BMI), waist circumference, percent body fat, or visceral fat area, it is unclear which specific measure is best associated with mobility disability in oldest-old adults.

**Methods:**

Among 589 Chinese participants aged 85 years and older in a population-based cohort in Singapore, we measured waist circumference, computed BMI, estimated appendicular skeletal muscle mass, percent body fat, and visceral fat area using bioelectrical impedance analysis, and evaluated mobility disability using the Loco-Check questionnaire. We computed areas under the receiver operating characteristic curves (AUC_ROC_) to compare how well these measures discriminated between those with and without mobility disability. Logistic regression models were used to estimate the odds ratios (OR) and 95% confidence intervals (CI) for the associations between obesity defined by these measures and mobility disability.

**Results:**

Compared to BMI, which had an AUC_ROC_ (95% CI) of 0.68 (0.64–0.72) for the discrimination of mobility disability, only visceral fat area had a significantly higher discriminative performance [AUC_ROC_ (95% CI) of 0.71 (0.67–0.75) (P_adjusted_ = 0.002)]. The optimal cut-offs of visceral fat area for the discrimination of mobility disability were ≥ 104 cm^2^ in men and ≥ 137 cm^2^ in women. In fully adjusted models, only obesity defined by visceral fat area was significantly associated with mobility disability [OR (95% CI) of 2.04 (1.10–3.77)]; obesity defined by the other measures were not associated with mobility disability after adjusting for visceral fat.

**Conclusion:**

In oldest-old adults, visceral fat area was the best discriminator for obesity associated with mobility disability.

**Supplementary Information:**

The online version contains supplementary material available at 10.1186/s12877-021-02226-6.

## Background

Obesity is defined as an abnormal or excessive accumulation of fat that presents a risk to health [1]. In older adults, numerous studies have shown that obesity affects not only morbidity and mortality [2–8], but also quality of life and risk of institutionalization [2, 6–8]. Obesity, however, is also recognized as a heterogeneous disorder [9, 10]. Obese individuals are known to vary in their body fat distribution, and increasing evidence suggests that the regional distribution of adipose tissue might be more important than the total amount of body fat [9, 10]. Body fat tissue has traditionally been thought to be distributed across two main compartments – subcutaneous fat, and visceral fat [11]. These two different fat depots have been shown to have disparate functions, biochemical features, and metabolic characteristics [11, 12].

Unfortunately, the gold-standard methods for accurately identifying and measuring adiposity in these compartments, such as magnetic resonance imaging (MRI) and computed tomography (CT), require the use of sophisticated equipment that are not always readily available [2, 8]. As such, the World Health Organization (WHO), epidemiological studies, and clinicians typically utilize body mass index (BMI) as a surrogate for overall adiposity in clinical and public health practice [1–3]. BMI, which is a composite index of total body weight accounting for height [3], is easy and inexpensive to measure [2, 8], and has been shown to correlate reasonably well with overall adiposity in younger and middle-aged adults [7, 8].

However, BMI cannot differentiate between fat mass and lean muscle (fat-free) mass [2–5, 8]; as such, many studies have since found it to be a poor measure of overall adiposity in older adults [2–5, 8]. In addition to BMI, waist circumference has also been widely used as a surrogate measure for total abdominal adiposity in the assessment of body composition in older adults [3, 4, 6, 7, 13]; however, this measure is unable to differentiate between the subcutaneous and the visceral fat within the abdominal region [11]. Recently, bioelectrical impedance analysis (BIA) has emerged as a simple, rapid, inexpensive, and non-invasive diagnostic tool for the assessment of body composition [2, 14]. BIA utilizes differences in resistance between the fat and lean components of the body to provide an estimate for fat-free mass, total body fat, and more recently, for abdominal visceral fat [11].

In older adults, obesity is known to be associated with impaired function and physical disability [2–4, 6–8]. However, it remains unclear whether it is total body fat, subcutaneous fat, or visceral fat that is specifically related to mobility disability in older adults. Knowledge about the specific measure of obesity that is best associated with mobility disability in older adults could be applied in the screening of at-risk individuals, and in the design of specific interventions targeted at reducing obesity for attenuating the risk of mobility disability in the older adult population.

In our study, we evaluated the performance of four clinical measures of obesity – namely BMI, waist circumference, percent body fat, and visceral fat area – in discriminating between individuals with and without mobility disability among community-dwelling participants aged 85 years and older. The oldest-old (85 years and above) constitute 7% of the world’s 65 years-and-over population [15], and although they are generally recognized as the fastest growing segment of the population in many countries, they remain under-represented in ageing studies [16].

## Methods

### Study population

We used data from the SG90 study, which is nested within the population-based Singapore Chinese Health Study [17] (Supplementary Figure S1, Additional file 1). The latter is an on-going prospective cohort study designed to evaluate the genetic, dietary, and environmental determinants of chronic diseases in Chinese adults living in Singapore. Detailed descriptions of the study have been reported previously [17]. In brief, 63,257 participants (27,959 men and 35,298 women) aged 45–74 (mean 53) years old were enrolled between April 1993 and December 1998. Study participants belonged to either of the two major dialect groups of Chinese in Singapore (Hokkiens or Cantonese), and they were permanent residents or citizens who resided in government-built housing estates – where 86% of all Singaporeans resided during that period. After recruitment, the participants were re-contacted for follow-up interviews in 1999–2004 (phone), 2006–2010 (phone), and 2014–2017 (face-to-face) (Supplementary Figure S1, Additional file 1). The study was approved by the Institutional Review Board at the National University of Singapore, and written informed consent was obtained from all study participants.

There were 1305 participants who had hitherto participated in all three of the follow-up interviews, and who were also 85 years and older during the recruitment period of this study from July 2017 to August 2018. In this SG90 sub-cohort, we recruited the first 1000 consenting participants for in-person interviews that examined specific measures relevant to ageing, such as cognitive performance, quality of life, functional independence, etc. (Supplementary Figure S1, Additional file 1). During these interviews, we also asked participants for their sociodemographic characteristics and history of physician-diagnosed diseases that included diabetes, stroke, coronary artery disease, arthritis, and osteoporosis/hip fracture. We used the Singapore-modified Mini-Mental State Examination (SM-MMSE) [18] to screen participants for cognitive impairment. We also measured each participant’s handgrip strength and usual gait speed in line with methods described by the 2019 consensus update from the Asian Working Group for Sarcopenia (AWGS 2019), and then used the recommended cut-off values to detect cases of sarcopenia [19].

### Assessment of the four measures of obesity

Five hundred and eighty-nine of these participants additionally consented to undergo measurements for height, weight, and waist circumference, as well as to an assessment of body composition using bioelectrical impedance analysis (BIA). Each participant was visited by a trained interviewer and had their height measured to the nearest 1 cm using a stiff, self-retracting, metallic tape measure, and their weight measured to the nearest 0.1 kg with a Soehnle Exacta Comfort digital weighing scale (Model S63315 PSD). BMI (kg/m^2^) was computed as weight divided by height squared. Waist circumference was measured by positioning a soft, flexible, plastic tape measure at a level that was 2.5 cm (1 in.) above the participant’s navel [20].

We used the InBody S10 body composition analyser (Biospace Co. Ltd., Seoul, South Korea) to assess body composition through direct segmental multi-frequency bioelectrical impedance analysis (DSM-BIA). Each subject was asked to maintain a supine posture for at least 10 to 15 min before measurements were commenced. Eight tetrapolar adhesive electrodes (two for each hand and foot) were then attached to the participant, and they were used to obtain 30 impedance measurements across six frequencies (1, 5, 50, 250, 500, and 1000 kHz) as well as 15 reactance and phase angle measurements across three frequencies (5, 50, and 250 kHz) at each of five body segments (left arm, right arm, trunk, left leg, and right leg). These resulting measurements were subsequently used to estimate appendicular skeletal muscle mass [19], percent body fat, and visceral fat area for each participant.

### Assessment of the mobility disability outcome

During the in-person interviews, participants were assessed for mobility disability using the Loco-Check questionnaire. This questionnaire was originally designed by the Japanese Orthopaedic Association as a self-reported checklist comprising seven physical activities related to daily living to screen for locomotive syndrome – defined as a condition of reduced mobility due to impairment of the locomotive organs [21]. Subsequent studies have shown that the Loco-Check questionnaire is also useful in estimating the extent of overall physical dysfunction in older adults [22, 23]. Participants were scored according to the number of activities that they reported having difficulty with, and those with a total score of four or more were considered to have mobility disability [24].

### Statistical analyses

We first evaluated which of the four measures of obesity had the best performance in discriminating between oldest-old adults with and without mobility disability through in-sample receiver operating characteristic (ROC) analysis. In this analysis, we first constructed a classification model for each of the four obesity measures using a logistic regression model which included sex and the respective obesity measure on a continuous linear scale as the exposures of interest, and mobility disability as the outcome of interest. This classification model was trained on the entire dataset, and its output (the estimated probability of mobility disability) was then tested against the observed outcome of mobility disability for every participant in the dataset. Using the ‘roctab’ and ‘roccomp’ commands in Stata [25], in-sample ROC curves were created by plotting the true positive rate (sensitivity) against the false positive rate (1 − specificity) as the discrimination threshold of the classification model was varied. Finally, we compared the areas under the in-sample ROC curves (in-sample AUC_ROC_) for waist circumference, percent body fat, and visceral fat area against that of BMI as a reference using an algorithm by DeLong et al. [26] through the ‘rocgold’ command in Stata [25], and further adjusted for multiple comparisons using Bonferroni correction. To further evaluate how these models might generalize to new cases, and to obtain more realistic estimates of their predictive performance, we also performed five-fold cross-validation using the ‘cvauroc’ package [27] in Stata [25]. In this cross-validation analysis, the dataset was first randomly partitioned into five equal-sized groups (folds). Then, for each of the four obesity measures, a classification model was trained on four of the five folds, and its output was then tested against the observed outcome for every participant in the remaining fold, creating a cross-validated ROC curve. This process was repeated five times for each obesity measure, until each of the folds had been used as the held-out test set, and five different cross-validated ROC curves had been generated. The mean area under these five cross-validated ROC curves was then reported for each obesity measure. As a sensitivity analysis to account for possible non-linear relationships between the obesity measures and mobility disability, we repeated both the in-sample and cross-validated ROC analyses, but modelled each obesity measure with a restricted cubic spline with three knots at its 10th, 50th, and 90th percentiles [28] using the ‘mkspline’ command in Stata [25]. We also evaluated the statistical significance of the non-linear spline term in each logistic regression model to assess for the presence of a non-linear association. For each obesity measure, we used the ‘roccomp’ command to compare the area under the in-sample ROC curve of the continuous linear model against that of its respective spline-transformed counterpart.

After visceral fat area had been identified as the measure of obesity with the highest discriminative performance, optimal cut-off points for men and women were derived separately by selecting the points with the highest Youden’s J statistic [29, 30] that also had at least 50% sensitivity and 50% specificity in sex-specific ROC curves. These cut-off points were used to define obesity by visceral fat area in a binary fashion. For additional comparisons, previously established cut-off points were also used to define cases of obesity through BMI (cut-off point of 27.5 kg/m^2^) [31], waist circumference (cut-off point of 90 cm for men and 80 cm for women) [32], and percent body fat (cut-off point of 30% for men and 40% for women) [33, 34]. Multivariable logistic regression models were used to compute odds ratios (OR) and 95% confidence intervals (CI) for the association between these four definitions of obesity and mobility disability. In Model 1, we adjusted for age, sex, and level of education (no formal education, primary, secondary and above). In Model 2, we additionally adjusted for other chronic conditions that could have contributed to mobility disability in older adults, such as diabetes, stroke, coronary artery disease, arthritis, osteoporosis/hip fracture, cognitive impairment, and sarcopenia. In Model 3, we further adjusted for the three respective measures (BMI, waist circumference, percent body fat, or visceral fat area) that were not utilized in that particular definition of obesity as continuous variables [35]. All statistical analyses were conducted using Stata/SE 14.2 software [25] and R version 3.5.3 software [36]. All *P*-values presented were two-sided, and *P* < 0.05 was considered statistically significant.

## Results

Of the 589 oldest-old participants included in our analyses, 356 (60.4%) of them were women. The mean [standard deviation (SD)] age of the participants was 88 (2.4) years, and their ages ranged from 85 to 97 years. All the four measures of obesity had moderate to high correlations with one another; the Pearson’s correlation coefficients (r) for pairwise correlations ranged from 0.59 (between percent body fat and waist circumference) to 0.94 (between percent body fat and visceral fat area) (Table 1). 284 (48.2%) participants were defined as having mobility disability using the Loco-Check questionnaire.

**Table 1:** Pearson’s correlation coefficients for the pairwise correlations among the four measures of obesity

	Body mass index	Waist circumference	Percent body fat	Visceral fat area
Body mass index	1.00			
Waist circumference	0.82	1.00		
Percent body fat	0.74	0.59	1.00	
Visceral fat area	0.82	0.71	0.94	1.00

**Fig. 1:**
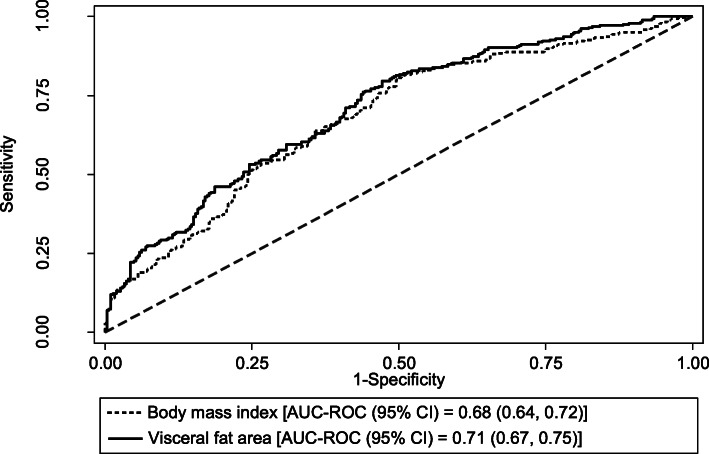
Discriminative performance of visceral fat area vs. body mass index. Legend: In-sample ROC curves for the performance of (1) body mass index and (2) visceral fat area in discriminating between oldest-old adults with and without mobility disability, adjusted for sex. ROC: receiver operating characteristic; AUC-ROC: area under the ROC curve; CI: confidence interval

When adjusted for sex, BMI had an in-sample AUC_ROC_ (95% CI) of 0.68 (0.64, 0.72) for discriminating between oldest-old adults with and without mobility disability, (Table 2) (Fig. 1). Waist circumference and percent body fat initially appeared to have nominally higher in-sample discriminative performances when compared to BMI; however, these differences were no longer statistically significant after correction for multiple comparisons (P_adjusted_ ≥ 0.075) (Table 2). Only visceral fat area, which had an in-sample AUC_ROC_ (95% CI) of 0.71 (0.67, 0.75), had a significantly higher in-sample discriminative performance when compared to BMI (P_adjusted_ = 0.002) (Table 2) (Fig. 1). The discriminative performances of the four obesity measures remained mostly unchanged when they were validated through five-fold cross-validation (Supplementary Table S1, Additional file 1).

**Table 2:** In-sample performance of the four obesity measures in discriminating between oldest-old adults with/without mobility disability, adjusted for sex

Measure of obesity	In-sample AUC_**ROC**_ (95% CI)	***P***-value	Bonferroni-adjusted ***P***-value
Linear models ^a^			
Body mass index	0.68 (0.64, 0.72)	Ref.	Ref.
Waist circumference	0.70 (0.65, 0.74)	0.040	0.12
Percent body fat	0.70 (0.66, 0.74)	0.025	0.075
Visceral fat area	0.71 (0.67, 0.75)	< 0.001	0.002
Restricted cubic spline models ^b^			
Body mass index	0.68 (0.64, 0.73)	Ref.	Ref.
Waist circumference	0.70 (0.65, 0.74)	0.28	0.84
Percent body fat	0.70 (0.66, 0.74)	0.079	0.24
Visceral fat area	0.71 (0.67, 0.75)	0.024	0.072

*AUC*_*ROC*_ area under the receiver operating characteristic curve, *CI* confidence interval

^a^ Obesity measures were modelled on a continuous linear scale

^b^ Obesity measures were modelled with a restricted cubic spline with three knots at its 10th, 50th, and 90th percentiles

Earlier studies had reported non-linear relationships between measures of obesity like BMI and waist circumference on the risk for adverse outcomes such as mobility disability [37], physical frailty [38, 39], colon cancer [40], and mortality [41, 42]. To account for possible non-linear associations between the four obesity measures and mobility disability, and to evaluate any effects that these non-linearities might have had on their discriminative performances, we conducted a sensitivity analysis where the four obesity measures were modelled with restricted cubic splines (Table 2). There was some evidence of a non-linear association between the obesity measure and mobility disability for all four measures of obesity (P for non-linearity = 0.024, 0.023, 0.065, and 0.050 for BMI, waist circumference, percent body fat, and visceral fat area respectively). Nevertheless, we found that these restricted cubic spline models did not have significantly different in-sample discriminative performances when compared to their respective linear counterparts (*P* = 0.73, 0.88, 0.080, and 0.45 for the difference between the BMI, waist circumference, percent body fat, and visceral fat area models respectively) (Table 2). Furthermore, the overall trend of the discriminative performances across the four spline-transformed obesity measures remained largely unchanged in both in-sample (Table 2) as well as cross-validated ROC analyses (Supplementary Table S1, Additional file 1).

In this population of the oldest-old, women had a higher mean (SD) visceral fat area of 137.23 (45.65) cm^2^, compared to a mean (SD) visceral fat area of 99.73 (41.19) cm^2^ in men. The prevalence of mobility disability was also higher in women (60.1%) than in men (30.0%). In men, the optimal cut-off point for discriminating between individuals with and without mobility disability using visceral fat area was ≥104 cm^2^; this cut-off point gave an in-sample sensitivity of 60.0% and an in-sample specificity of 65.0% (Youden’s J statistic of 0.25). For women, the optimal cut-off point for visceral fat area was ≥137 cm^2^; this cut-off point gave an in-sample sensitivity of 60.8% and an in-sample specificity of 59.9% (Youden’s J statistic of 0.21).

Based on these sex-specific cut-off points, 286 (48.6%) participants were defined as being viscerally obese (Table 3). Compared to their counterparts, participants in the viscerally obese group were more likely to be women, be less educated, and to have a history of arthritis (Table 3). They were also more likely to have higher BMI values, larger waist circumferences and higher percent body fat measurements (Table 3). Interestingly, we also noticed that the participants in the viscerally obese group had a significantly lower prevalence of sarcopenia [65.6% vs. 81.5% respectively (*P* < 0.001)] (Table 3). This prompted us to investigate the odds of sarcopenia in the viscerally obese group compared to their non-obese counterparts. Indeed, once the other differentiating factors between these two groups (especially BMI, waist circumference, and percent body fat) had been adjusted for in a logistic regression model, the initial crude association between visceral obesity and sarcopenia [crude OR (95% CI) of 0.43 (0.30, 0.63)] was attenuated and no longer of statistical significance [adjusted OR (95% CI) of 0.61 (0.24, 1.52)]. Viscerally obese participants did not have a lower likelihood for sarcopenia after adjusting for other differentiating factors.

**Table 3:** Characteristics of viscerally obese versus non-viscerally obese participants

	Not viscerally obese	Viscerally obese	***P***-value
	(***N*** = 303)	(***N*** = 286)	
Mean visceral fat area (SD) / cm^2^	85.66 (26.22)	161.31 (31.45)	< 0.001
Mean body mass index (SD) / kg/m^2^	20.66 (2.59)	26.02 (3.55)	< 0.001
Mean waist circumference (SD) / cm	81.05 (8.57)	93.97 (7.96)	< 0.001
Mean percent body fat (SD) / %	30.64 (7.16)	43.60 (5.80)	< 0.001
Mobility disability (total score ≥ 4)	112 (37.0%)	172 (60.1%)	< 0.001
Mean Loco-Check score (SD)	3.00 (1.66)	4.04 (1.79)	< 0.001
Mean age at test (SD) / years	87.73 (2.45)	87.88 (2.32)	0.45
Men	134 (44.2%)	99 (34.6%)	0.017
Level of education			< 0.001
No formal education	99 (32.7%)	141 (49.3%)	
Primary	153 (50.5%)	116 (40.6%)	
Secondary and above	51 (16.8%)	29 (10.1%)	
Diabetes	68 (22.4%)	81 (28.3%)	0.10
Stroke	23 (7.6%)	28 (9.8%)	0.34
Heart diseases	56 (18.5%)	61 (21.3%)	0.39
Arthritis	71 (23.4%)	96 (33.6%)	0.006
Osteoporosis / hip fracture	40 (13.2%)	44 (15.4%)	0.45
Cognitive impairment	62 (20.5%)	78 (27.3%)	0.052
Sarcopenia (AWGS 2019)	247 (81.5%)	187 (65.6%)	< 0.001

*Legend*: Continuous variables were presented as mean (SD), while categorical variables were presented as N (%). *SD* standard deviation, *AWGS 2019* Asian Working Group for Sarcopenia 2019 consensus update

Compared to their leaner counterparts after adjustments for age, sex, level of education, and other chronic conditions related to mobility disability (including sarcopenia) (Model 2), those with visceral obesity were associated with a significantly increased risk for mobility disability [OR (95% CI) of 2.36 (1.61, 3.47)] (Table 4). Even after adjusting for BMI, waist circumference, and percent body fat (Model 3), visceral obesity remained associated with a significantly increased risk for mobility disability [OR (95% CI) of 2.04 (1.10, 3.77)] (Table 4). We then further investigated the association between obesity defined by the other measures and the odds of mobility disability. After adjusting for visceral fat area and the other obesity measures (Model 3), obesity defined by BMI [OR (95% CI) of 1.43 (0.70, 2.94)], by waist circumference [OR (95% CI) of 0.96 (0.56, 1.62)] and by percent body fat [OR (95% CI) of 1.29 (0.69, 2.40)] were no longer associated with an increased risk for mobility disability (Table 4).

**Table 4:** Associations between the four definitions of obesity and odds of mobility disability

	N (%)	Model 1 ^**a**^	Model 2 ^**b**^	Model 3 ^**c**^
		OR (95% CI)	OR (95% CI)	OR (95% CI)
Obesity defined by visceral fat area				
Not obese	303 (51.4%)	Ref.	Ref.	Ref.
Obese	286 (48.6%)	2.38 (1.68, 3.38)	2.36 (1.61, 3.47)	2.04 (1.10, 3.77)
Obesity defined by body mass index				
Not obese	505 (85.7%)	Ref.	Ref.	Ref.
Obese	84 (14.3%)	2.22 (1.33, 3.72)	2.57 (1.38, 4.79)	1.43 (0.70, 2.94)
Obesity defined by waist circumference				
Not obese	218 (37.0%)	Ref.	Ref.	Ref.
Obese	371 (63.0%)	1.85 (1.27, 2.68)	1.61 (1.06, 2.44)	0.96 (0.56, 1.62)
Obesity defined by percent body fat				
Not obese	257 (43.6%)	Ref.	Ref.	Ref.
Obese	332 (56.4%)	2.07 (1.45, 2.94)	2.13 (1.44, 3.13)	1.29 (0.69, 2.40)

*OR* odds ratio, *CI* confidence interval

^a^ Model 1: adjusted for age, sex, level of education

^b^ Model 2: adjusted for Model 1 and diabetes, stroke, heart diseases, arthritis, osteoporosis/hip fracture, cognitive impairment, sarcopenia

^c^ Model 3: adjusted for Model 2 and body mass index, waist circumference, percent body fat, visceral fat area

## Discussion

Our study compared the performance of four measures of obesity – namely BMI, waist circumference, percent body fat, and visceral fat area – in discriminating between community-dwelling oldest-old Chinese adults with and without mobility disability, and showed that visceral fat area had the highest discriminative performance. We further determined optimal cut-off points of ≥ 104 cm^2^ in men and ≥ 137 cm^2^ in women for the discrimination of oldest-old adults with mobility disability. Finally, we showed that obesity defined by visceral fat area remained consistently associated with significantly increased risks for mobility disability even after adjustments for chronic conditions like sarcopenia, and other measures of obesity like BMI, waist circumference, and percent body fat. Our findings provided evidence implicating the role of visceral fat in mobility disability among the oldest-old in the population.

Although BMI has been shown to correlate reasonably well with overall adiposity in younger and middle-aged adults [7, 8], many studies have since found that BMI is actually a poor measure of overall adiposity in older adults [2–5, 8]. The inability of BMI to accurately reflect the true extent of adiposity in older adults has been proposed to be caused by several different factors: First, BMI does not account for the fact that older adults lose height as they age, thus leading to overestimated values within these populations [2–4, 8]. Second, BMI cannot differentiate between fat mass and (fat-free) lean muscle mass, and it is thus unable to account for the age-dependent loss of lean body mass that commonly occurs in older adults [2, 3, 8]. Third, BMI is unable to distinguish between peripheral and visceral obesity [2, 4]. This last point is particularly pertinent to older adults as ageing is known to be associated with a decrease in peripheral and subcutaneous fat, while being associated with an increase in visceral fat [2–4].

There are few studies that have examined the association between waist circumference measurements and various aspects of disability in oldest-old adults. The Vitality 90+ Study in Finland studied 153 men and 416 women aged 90–91 years, and reported that when both BMI and waist circumference were included in the same model, only waist circumference remained significantly associated with an increased risk for disability in activities of daily living [OR (95% CI) of 3.17 (1.51, 6.68) for the highest compared to the lowest tertile of waist circumference] [43]. The Chinese Longitudinal Healthy Longevity Study (CLHLS), which included 2303 men and 3192 women aged 80 years and above living in China, reported that when compared to the lowest tertile, participants in the highest tertile of waist circumference also had a greater risk for disability in activities of daily living [OR (95% CI) of 2.01 (1.44, 2.82)] [44]. Yet another study conducted among 200 community-dwelling older adults aged 50 years and above in Singapore showed that when compared to BMI and percent body fat, sarcopenic obesity defined by waist circumference was best associated with poorer muscle function [34]. This finding was consistent with numerous other studies that had also found that waist circumference was more significantly associated with disability than BMI in younger populations of older adults [45–54].

Although waist circumference has been shown to be easy to measure in older adults, and strongly correlated to both total body fat and visceral fat as measured by CT [3, 7], the use of different anatomical landmarks to measure waist circumference might lead to different classifications of abdominal obesity [35, 55], and this might be more problematic in our population of oldest-old adults [20]. The other limitation inherent to waist circumference measurements is the inability to differentiate between the subcutaneous fat and the visceral fat within the abdominal region [11]. As such, our study meaningfully expanded upon the findings of those prior studies by showing that it was the amount of visceral fat, and not subcutaneous fat, that was associated with mobility disability in oldest-old adults. To our best knowledge, this is the first study that has investigated the association between visceral fat area and the risk of mobility disability in the older adult population.

The biological plausibility of our findings could be explained by the anatomical, cellular, molecular, and physiological differences between subcutaneous adipose tissue and visceral adipose tissue [9]. For example, visceral adipose tissue is known to contain a greater percentage of large adipocytes, and a larger number of inflammatory and immune cells than subcutaneous adipose tissue [9]. Furthermore, the adipocytes in visceral adipose tissue are also known to be more metabolically active and more insulin-resistant [9]. Hence, not unexpectedly, compared to general obesity, visceral obesity has also been shown to be more closely associated with a wide variety of chronic diseases such as type-2 diabetes, atherogenic dyslipidemia, cardiovascular disease, hypertension, specific cancers, obstructive sleep apnea, and metabolic syndrome [10]. In this study, we further showed that visceral obesity could also have significant implications on mobility disability – defined as dependence in carrying out physical activities related to daily living due to reduced mobility [21]. Among the oldest-old, this is undoubtedly an important ageing outcome.

In our oldest-old population, we found that women had a higher mean visceral fat area and a higher optimal cut-off point for visceral obesity when compared to men. These findings are discrepant from what is known in younger adult populations, where women have been shown to have less visceral fat [56, 57] and lower cut-off points for risk of heart disease, type-2 diabetes, and certain cancers [56]. Although age is known to be associated with an increase in visceral fat, more recent studies have since found sex-specific differences in the rate of increase in these age-related changes. For example, a study in the United States reported that between the 3rd and 7th decades of life, men had over a 200% increase in visceral fat, whereas women had over a 400% increase [56]. Similarly, another study in Japan showed that between 40 and 79 years, visceral fat area increased by 42.9% in men compared with 65.3% in women [57]. This greater rate of age-related increase in visceral fat in women compared to men might possibly explain the higher values seen in our population of oldest-old women. Finally, since having higher visceral fat areas has been associated with increased risk of severe morbidity and/or mortality earlier in life [10], our finding of a higher visceral fat area in women than in men in this population of the oldest-old could also be explained by a relatively higher survival bias in men with lower visceral fat than in women.

Since the obesity measures and outcome of mobility disability were assessed at the same time in the cross-sectional design of this study, we acknowledge that reverse causality cannot be excluded, and that we are unable to conclusively determine the temporal relationship between obesity and mobility disability. Another limitation concerned our definition of mobility disability, which was assessed for using the Loco-Check questionnaire – a self-reported checklist [21]. In older adults, studies have noted discrepancies or contrasting results when functional limitations were assessed through self-report, as opposed to when these limitations were assessed by means of objective performance-based tests [58–61]. These studies found that affective functioning and personality traits [58], such as perception of physical competence, mastery or personal control, and levels of depressive symptomatology [59], affected the discrepancies between self-reported and performance-based functional limitations. Nevertheless, in spite of these discrepancies, these studies still reported moderate [58] to strong [60, 61] correlations between self-reported and performance-based functional limitations. One other potential limitation of our study concerned the generalizability of our percent body fat and visceral fat area measurements, as well as the applicability of our visceral fat area cut-off points, which were all estimated through DSM-BIA. Studies have suggested that, among other factors, biological differences between different ethnic populations might affect the relationship between electrical impedance and body composition [14], leading to inaccurate estimates of both fat and lean muscle mass. As such, our findings need to be replicated in other oldest-old populations to validate their generalizability. Going forward, it will also be important to investigate whether these findings apply to younger, general populations of older adults (i.e., those aged 65 years and above).

## Conclusions

Our study found that when compared to other common measures of obesity, visceral fat area had the highest performance in discriminating between oldest-old adults with and without mobility disability. We also showed that obesity defined by visceral fat area remained consistently associated with significantly increased risks for mobility disability even after adjusting for chronic conditions like sarcopenia, and other measures of obesity like BMI, waist circumference, and percent body fat. Our findings are aligned with studies which implicate the putative role of visceral fat with risk of chronic diseases among older adults, and they provide support for measuring visceral fat using BIA in the screening of at-risk oldest-old individuals in the community. Clinical interventions targeting the reduction of visceral fat may also decrease the likelihood of mobility disability in the oldest-old population.

## Supplementary Information

**Additional file 1: Supplementary Table S1.** Cross-validated performance of the four obesity measures in discriminating between oldest-old adults with/without mobility disability, adjusted for sex. Five-fold cross-validated performance of the four measures of obesity (modelled both linearly and with restricted cubic splines) in discriminating between oldest-old adults with and without disability after adjustment for sex. **Supplementary Figure S1.** Flowchart of the Singapore Chinese Health Study and the SG90 sub-cohort. Flowchart detailing the Singapore Chinese Health Study, its three follow-up interviews, as well as the SG90 sub-cohort.

## Data Availability

The datasets generated and/or analysed during the current study may be available from the corresponding author on reasonable request.

## Abbreviations

AUC_ROC_: Area under the receiver operating characteristic curve
BIA: Bioelectrical impedance analysis
BMI: Body mass index
CI: Confidence interval
CLHLS: Chinese Longitudinal Healthy Longevity Study
CT: Computed tomography
DSM-BIA: Direct segmental multi-frequency bioelectrical impedance analysis
MRI: Magnetic resonance imaging
OR: Odds ratio
ROC: Receiver operating characteristic
SD: Standard deviation
SM-MMSE: Singapore-modified Mini-Mental State Examination
WHO: World Health Organization
