# Supplementary material for: Visceral fat area is the measure of obesity best associated with mobility disability in community dwelling oldest-old Chinese adults
Source: BMC Geriatr. 2021 Apr 28;21:282. doi: 10.1186/s12877-021-02226-6 (PMC8082923; doi:10.1186/s12877-021-02226-6)
Supplement: Supplementary file 1 — Additional file 1: Supplementary Table S1. Cross-validated performance of the four obesity measures in discriminating between oldest-old adults with/without mobility disability, adjusted for sex. Five-fold cross-validated performance of the four measures of obesity (modelled both linearly and with restricted cubic splines) in discriminating between oldest-old adults with and without disability after adjustment for sex. Supplementary Figure S1. Flowchart of the Singapore Chinese Health Study and the SG90 sub-cohort. Flowchart detailing the Singapore Chinese Health Study, its three follow-up interviews, as well as the SG90 sub-cohort. [file 12877_2021_2226_MOESM1_ESM.docx]

**Visceral fat area is the measure of obesity best associated with mobility disability in community dwelling oldest-old Chinese adults**

Kevin Yiqiang Chua ^1,2^, Xinyi Lin ^3,4^, Yeli Wang ^5^, Yap-Seng Chong ^6,7^, Wee-Shiong Lim ^8^, Woon-Puay Koh ^9^

1. Integrative Sciences and Engineering Programme, NUS Graduate School, National University of Singapore, Singapore
2. Saw Swee Hock School of Public Health, National University of Singapore, Singapore
3. Centre for Quantitative Medicine, Duke-NUS Medical School, Singapore
4. Singapore Clinical Research Institute, Singapore
5. Health Services and Systems Research, Duke-NUS Medical School, Singapore
6. Department of Obstetrics & Gynaecology, Yong Loo Lin School of Medicine, National University of Singapore, National University Health System, Singapore
7. Singapore Institute for Clinical Sciences, Agency for Science Technology and Research (A*STAR), Singapore
8. Department of Geriatric Medicine, Institute of Geriatrics and Active Aging, Tan Tock Seng Hospital, Singapore
9. Healthy Longevity Translational Research Programme, Yong Loo Lin School of Medicine, National University of Singapore, Singapore

**Corresponding Author:**

Woon-Puay Koh. Healthy Longevity Translational Research Programme, Yong Loo Lin School of Medicine, National University of Singapore. S9, 5 Science Drive 2, Level 11, Singapore 117545, Singapore. Phone: (65) 6601 6864; Email: [kohwp@nus.edu.sg](mailto:kohwp@nus.edu.sg).

**Supplementary Table S1:** Cross-validated performance of the four obesity measures in discriminating between oldest-old adults with/without mobility disability, adjusted for sex.

| **Measure of obesity** | **Mean cross-validated AUC_ROC_ (95% bootstrap bias corrected CI)** |
| --- | --- |
| Linear models ^a^ |  |
| Body mass index | 0.68 (0.64, 0.73) |
| Waist circumference | 0.69 (0.65, 0.75) |
| Percent body fat | 0.70 (0.65, 0.75) |
| Visceral fat area | 0.70 (0.66, 0.76) |
| Restricted cubic spline models ^b^ |  |
| Body mass index | 0.68 (0.63, 0.73) |
| Waist circumference | 0.69 (0.65, 0.74) |
| Percent body fat | 0.70 (0.66, 0.75) |
| Visceral fat area | 0.71 (0.67, 0.76) |

**Legend:** AUC_ROC_: area under the receiver operating characteristic curve; CI: confidence interval.

^a^ Obesity measures were modelled on a continuous linear scale.

^b^ Obesity measures were modelled with a restricted cubic spline with three knots at its 10th, 50th, and 90th percentiles.


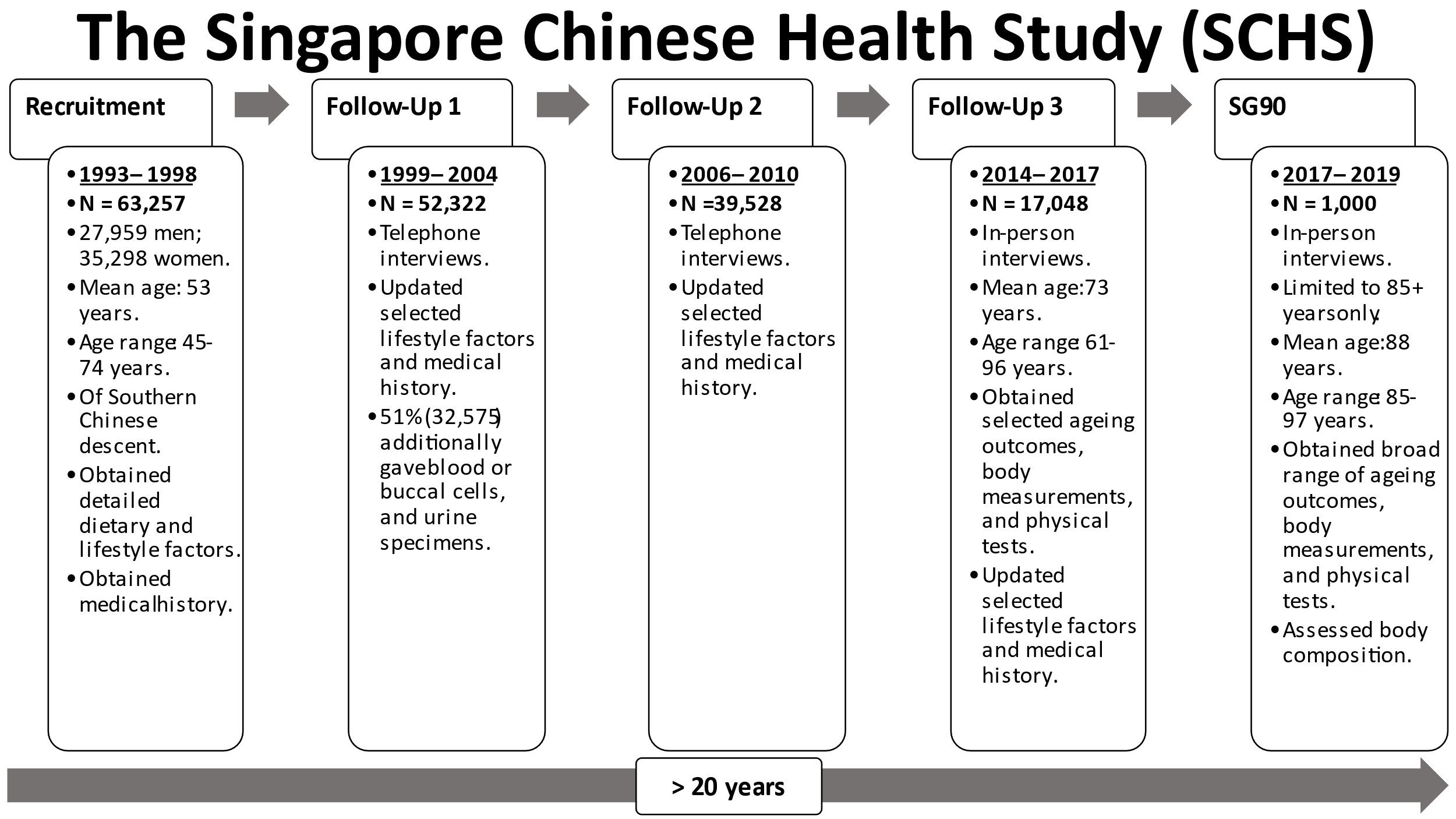


**Supplementary Figure S1:** Flowchart of the Singapore Chinese Health Study and the SG90 sub-cohort.
